# Improvements in Digestive Symptoms After Participation in an App-Based Chronic Digestive Disease Management Program: A Prospective Cohort Evaluation

**DOI:** 10.7759/cureus.66941

**Published:** 2024-08-15

**Authors:** Dena M Bravata, Hau Liu, Meghan M Colosimo, Alexander C Bullock, Erin Commons, Mark Pimentel

**Affiliations:** 1 Internal Medicine, Center for Primary Care and Outcomes Research, Stanford University School of Medicine, Stanford, USA; 2 Diabetes and Endocrinology, New York University (NYU) Grossman School of Medicine, New York, USA; 3 Data Science, Vivante Health, Chicago, USA; 4 Strategic Analytics, Vivante Health, Chicago, USA; 5 Nutrition, Vivante Health, Chicago, USA; 6 Gastroenterology, Cedars-Sinai Medical Center, Los Angeles, USA

**Keywords:** patient-reported outcomes, medical nutrition therapy, telemedicine, digestive disease, occupational health, digital disease management, chronic gastrointestinal symptoms

## Abstract

Background: Fewer than 20% of adults with chronic gastrointestinal (GI) symptoms have accessed care to evaluate or manage their symptoms. We sought to characterize whether adults with chronic GI symptoms would use an app for symptom monitoring and the effects of participation in a digitally delivered GI chronic care program.

Methods: We provided a digital digestive care management app to adults via their employer-sponsored benefits. We evaluated participants’ self-reported GI symptoms at baseline and between 30 and 90 days post-registration. GI symptoms (e.g., abdominal pain and constipation) were rated on a scale of 0 (no symptoms) to 4 (very severe symptoms).

Results: A total of 1936 participants were enrolled (75% female; 67% White, 11% Asian/Pacific Islander, 6% Hispanic, 7% Black; mean age: 43 years). Their most common GI conditions were irritable bowel syndrome (IBS), gastroesophageal reflux disease (GERD), and acid reflux. Participants of all genders and races reported statistically significant improvements in all symptoms between baseline and the end of the intervention (P < 0.05). At baseline, 79.5% of participants reported at least moderate GI symptom severity for at least one symptom. In contrast, at the end of the intervention, only 47.8% of participants reported moderate or severe symptoms, and 310 (16.0%) participants reported no symptoms. Participants who were scheduled with their care team reported greater symptom improvement than those who were not scheduled (P = 0.004). Participants reported feeling greater control of their health (83%), better management of their digestive symptoms (83%), increased happiness (76%), and greater productivity at work (54%).

Conclusion: Demographically diverse participants engaged with a digital digestive chronic care program and reported significant improvements in digestive symptom severity.

## Introduction

Chronic gastrointestinal (GI) disease burdens are enormous, accounting for substantial morbidity, mortality, and cost [1-4]. In the United States, GI conditions are the principal cause of 105 million ambulatory care visits, 14 million hospital admissions, 236,000 deaths, and $142 billion in total costs [3,4]. More than 40% of adults reported one or more digestive symptoms monthly, with abdominal pain or discomfort (21.8%), bloating or distension (15.9%), and diarrhea or loose stools (26.9%) being the most common [1]. More than 65% of adults with chronic GI issues rated their symptoms as moderate or severe in intensity, and the majority reported limitations in daily activities [1]. Employers bear a significant burden of excessive costs associated with GI conditions, given that chronic GI symptoms peak in mid-life and affect working adults [4]. Employees who report GI symptoms are more likely to miss work, be less productive, and quit their jobs than those who do not have chronic GI symptoms [5,6]. The average annual cost per employee with chronic GI symptoms varies widely from $13,948 for functional gastrointestinal disorders to $36,441 for ulcerative colitis and $62,735 for Crohn’s disease [4].

Unfortunately, historically, less than 20% of individuals with abdominal pain, bloating, or diarrhea consult a healthcare provider to evaluate and manage their symptoms [1]. Telemedicine offers the potential for increased access to GI specialty care. Unfortunately, no consensus exists on the best instruments to assess chronic GI symptoms. Among GI symptoms questionnaires that are most cited [7,8], many are cumbersome for patients to use frequently (i.e., include 20 or more items), assess symptoms other than common GI issues (e.g., pelvic floor symptoms), and have not been evaluated for use in virtual care settings. This lack of standardized chronic GI symptom trackers is a barrier to evaluating interventions for patients with chronic GI issues, especially virtual GI specialty care.

We evaluated the effects of a digital digestive chronic care program that included longitudinal symptom tracking, personalized medical nutrition therapy, health coaching, and targeted education on nine common GI symptoms collected via an app. Specifically, we sought to first characterize whether adults with chronic GI symptoms would use an app for symptom monitoring and, second, to assess the effects of participation in a digitally delivered chronic care program on GI symptom severity.

## Materials and methods

Recruitment

Adult participants across the United States were provided access to a digital digestive chronic care program called GIThrive (Vivante Health, Houston, Texas) through their employee benefits [9]. Employees were invited to join the program via employer-approved marketing materials through mailings, emails, and other outreach. Any employee could join, regardless of prior digestive diagnoses or symptoms. Recruitment materials highlighted the importance of digestive wellness to overall health and targeted individuals suffering from common GI symptoms such as heartburn, gas, bloating, and indigestion. Those participants who enrolled in the program between January 1, 2022, and November 28, 2023, who provided data tracking symptoms on the app between days 30 and 90 after registration, and who provided symptom data on more than one occasion were included in this evaluation. Participants were given free access to the digital digestive chronic care program but were not otherwise compensated for their participation.

Data collection

Participants were asked to complete an intake survey that included digestive symptoms at program initiation (baseline) and then track symptoms throughout the program. Surveys and symptom tracking were administered within the app. At baseline, users also provided demographics and their GI history, including previously diagnosed GI conditions.

GI Symptoms

At the baseline survey, participants provided information on their digestive symptoms over the last week. Participants tracked their symptoms during their care via the app to assess clinical progress and guide subsequent interventions. They were asked to rate nine common GI symptoms (abdominal pain, bloating, diarrhea, constipation, reflux, gas, nausea, vomiting, and loss of bowel control) on a five-point scale: 0 (no symptoms), 1 (mild symptoms), 2 (moderate symptoms), 3 (severe symptoms), and 4 (very severe symptoms). We computed individual scores for each of the nine symptoms and an overall digestive symptom score (0 to 36, computed as the sum of each of the scores) at baseline and the last recorded symptom tracking between 30 and 90 days. If participants recorded symptoms more than one time per day, we used the last data they provided.

Participant Experience

On day 28 of the program, participants were sent a five-item survey about their experience with the program. Four questions had three response options (yes, no, or not applicable): (1) “Since joining the program, I feel more in control of my health”; (2) “I’m happier since using the program”; (3) “Since joining the program, I feel more productive at work”; and (4) “The program has helped me better manage my digestive symptoms.” The fifth question asked, “Where would you have gone for digestive health care if you had not signed up for the program?”

Intervention

The digital digestive chronic management program has four key components: symptom tracking, personalized medical nutrition therapy, health coaching, and targeted education. The platform is available as a native app on iOS and Android devices and a web app on personal computers. It is compliant with HIPAA (Health Insurance Portability and Accountability Act) and SOC2 + HITRUST (Service Organization Control 2 + Health Information Trust Alliance).

Symptom Tracking

A key goal of the intervention is to facilitate easy symptom tracking so that users can see how their symptoms have changed over time. The care team evaluates how different foods and lifestyle elements (physical activity, stress, and sleep habits) impact symptoms. The app guides users with diet and lifestyle interventions to promote symptom reduction, and users can follow their progress in their app.

Personalized Medical Nutrition Therapy

Registered dietitians provide 40-minute, evidence-based [10-12], one-on-one medical nutrition therapy to participants both synchronously and asynchronously via telephonic, video, and instant messages in compliance with state(s) licensure based on the user’s primary concern. Common topics of discussion include nutritional monitoring and management of Crohn’s disease, ulcerative colitis, irritable bowel syndrome (IBS), ostomy nutrition, short bowel syndrome, diverticulitis, chronic constipation or diarrhea, gastroparesis, elimination diets, FODMAP (fermentable oligosaccharides, disaccharides, monosaccharides, and polyols) diet, food allergies and intolerances, SIBO (small intestinal bacterial overgrowth), and malnutrition.

Dietitians with expertise in counseling skills are recruited and trained for more than 80 hours on advanced topics in digestive health conditions and symptom management before initiating patient care. All dietitians received an additional 35 credit hours to obtain their low FODMAP certification. This care has been associated with clinically significant symptom reduction and identification of symptom triggers [13]. Dietitians use the platform’s educational resources, health coaches, on-demand nursing referrals, and external referrals as needed to optimize patient care.

Health Coaching

All participants have access to one-on-one telephonic, video, and instant message coaching to help with goals related to the management of their GI conditions, including stress management, mindfulness, healthy sleep, medication adherence, self-advocacy, physical activity, self-monitoring, coach-lead cognitive behavioral therapy, and assistance with the app.

Health coaches are trained to help users set goals, develop self-efficacy and self-advocacy, and utilize disease management resources. Specifically, they apply the Member Engagement Model, which includes motivational interviewing, positive psychology, and the transtheoretical model to drive behavior change. Coaches use digestive health-specific protocols to promote symptom relief, digestive condition treatment, and behavior change. They work closely with registered dietitians to establish and coordinate user-specific care plans.

Targeted Education

The program provides each user with a personalized care plan that includes targeted education on their conditions, symptoms, and supportive lifestyle interventions. Each time a user interacts with the app, they are reminded of the key interventions to drive clinical outcomes based on their clinical status. Additionally, users can avail themselves of resources like courses, articles, recipes, and weekly webinars

Escalation Protocol

If a user reports significant symptom acuity either through the app or to a member of the care team, nursing staff are available on call at all times to provide support, education, and triage to the appropriate level of care.

Statistical Analysis

We computed means at baseline for each of the outcomes of interest. We evaluated patterns of engagement, symptom clustering, and change in symptoms by ANOVA and considered P-values < 0.05 to be statistically significant. Analyses were performed using SPSS v29 (IBM Corp., Armonk, NY).

Human Subjects Approval

Given that all data were routinely collected as part of the condition management program, this protocol was considered exempt by the WCG IRB (VORD.00A, April 2023).

## Results

Participant characteristics

Overall, 1936 participants completed the registration process and were enrolled in the study. The average age of participants was 43.1 years (SD 11.5 years), 75% identified as female, 67% identified as White/Caucasian, 11% as Asian/Pacific Islander, 6% as Hispanic/Latinx, 7% as Black, and 7% as being of multiple races (Table 1).

**Table 1:** Baseline participant characteristics (N total = 1936) GI: Gastrointestinal; GERD: Gastroesophageal reflux disease.

Characteristics	Mean (%)
Age (years)	43.0 years (SD = 11.5 years)
Gender	
Female	1450 (74.9%)
Male	474 (24.5%)
Prefer not to disclose	12 (0.6%)
BMI (kg/m^2^)	28.5 (SD = 7.4)
<18.5 (underweight)	31 (1.6%)
18 to 25 (normal weight)	650 (33.6%)
25 to 30 (overweight)	599 (30.9%)
>30 (obese)	656 (33.9%)
Race/Ethnicity	
Caucasian/White	1292 (66.7%)
Asian/Pacific Islander	215 (11.1%)
Multiple	141 (7.3%)
African American/Black	139 (7.2%)
Latinx/Hispanic	116 (6.0%)
Native American	6 (0.3%)
Other	27 (1.4%)
GI conditions (reported by 714 participants)	
Irritable bowel syndrome	328 (16.9%)
GERD (acid reflux)	323 (16.7%)
Hemorrhoids	151 (7.8%)
Lactose intolerance	118 (6.1%)
Gastritis	104 (5.4%)
Hiatal hernia	75 (3.9%)
Gallstones	73 (3.8%)
Diverticulosis or diverticulitis	71 (3.7%)
Stomach or intestinal ulcers	52 (2.7%)
Small intestinal bacterial overgrowth (SIBO)	51 (2.6%)
Ulcerative colitis	37 (1.9%)
Celiac disease	32 (1.7%)
Gastroparesis	23 (1.2%)
Crohn’s disease	20 (1.0%)
Barrett’s esophagus	18 (0.9%)
GI bleeding	15 (0.8%)
Pancreatitis	15 (0.8%)
Colorectal cancer	11 (0.6%)
Other condition(s)	102 (5.3%)
None of these conditions	7 (0.4%)

On average, participants were overweight (mean BMI = 28.5 kg/m^2^, SD = 7.4 kg/m^2^; Table 1). Among those with a GI diagnosis, their most common GI conditions were IBS (16.9%) and gastroesophageal reflux disease (GERD) or acid reflux (16.7%), as shown in Table 1. Sixty percent of participants had two or more diagnosed GI conditions. Women were more likely to report gallstones (P = 0.008) and IBS (P = 0.001) than other genders. Participants with higher BMI were more likely to report diverticular disease (P = 0.003), gallstones (P < 0.001), GERD or acid reflux (P < 0.001), and small intestinal bacterial overgrowth (P = 0.007). At baseline, the total number of conditions was highly correlated with the total symptom score (r = 0.33, P < 0.001). At baseline, higher BMI was associated with more symptoms, especially diarrhea, nausea, bowel control, and reflux, as well as GI conditions (P < 0.001 for all associations).

Utilization of digestive care management

Participants used the app for various activities (e.g., symptom logging, reviewing educational materials, and interacting with their care team) during the study interval (between 30 and 90 days post-registration). Participants of all genders and races used the app similarly. Eighty-two percent scheduled at least one appointment with either a dietitian or a health coach between 30 and 90 days after registration. Participants with more GI conditions (P = 0.003) were more likely to remain engaged longer.

Clinical outcomes

GI Symptoms

**Figure 1:**
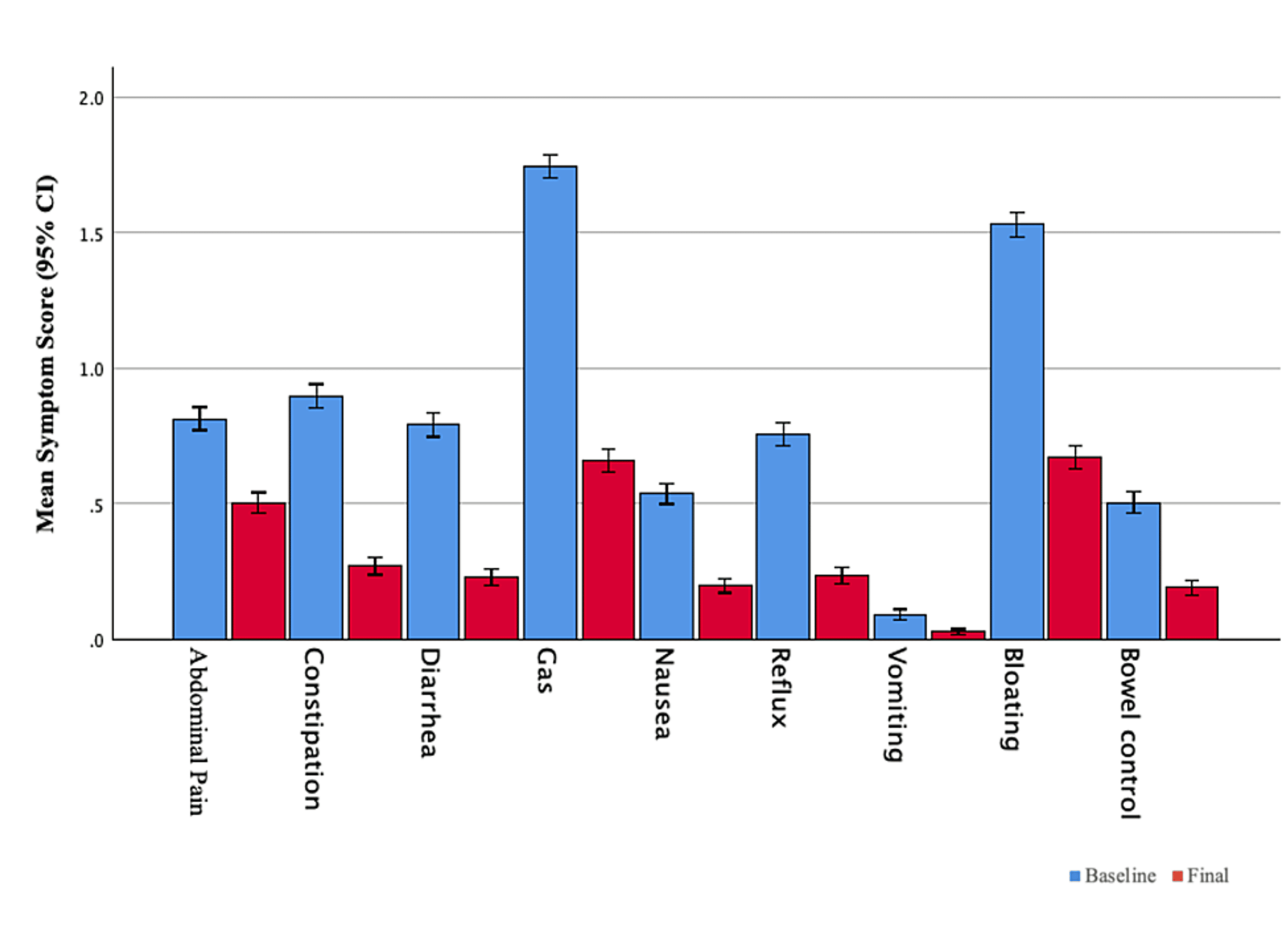
Change in GI symptom scores from baseline to the end of intervention Improvement in all symptoms was statistically significant (P < 0.05). GI: Gastrointestinal.

The most commonly reported symptoms at baseline were gas (93%), bloating (84%), constipation (55%), and abdominal pain (54%) (Figure 1).

Only 5% of participants reported vomiting. Participants reported combinations of GI symptoms in expected patterns (e.g., nausea often occurred with both abdominal pain and vomiting, abdominal pain co-occurred with bloating, and diarrhea with loss of bowel control). Baseline symptoms differed by gender and race with women reporting more abdominal pain, nausea, and constipation but less gas and Black/African Americans reporting more constipation than participants of other races.

Changes in GI Symptoms

**Figure 2:**
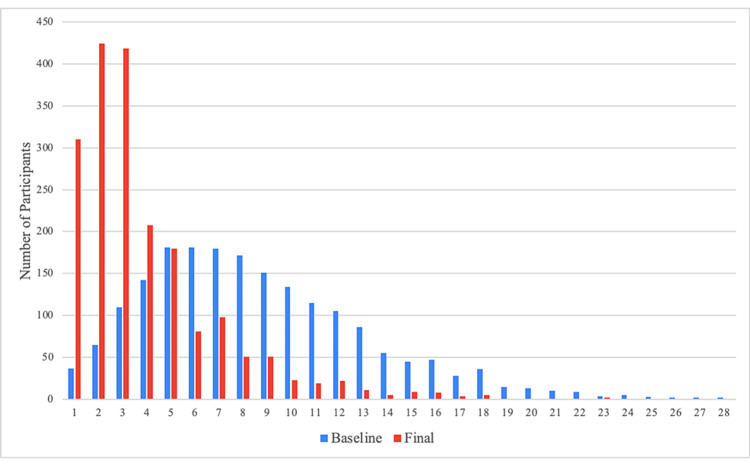
Change in total GI symptom score The total symptom score is on a scale from 0 to 36 (a sum of 0 to 4 across nine symptoms). GI: Gastrointestinal.

Participants reported statistically significant improvements in all symptoms between baseline and the end of the intervention (P < 0.05, Figure 1). At baseline, the mean total symptom score was 7.7 (SD 4.7), which improved at the end of the intervention to 3.0 (SD = 3.2, P < 0.001, Figure 2).

**Figure 3:**
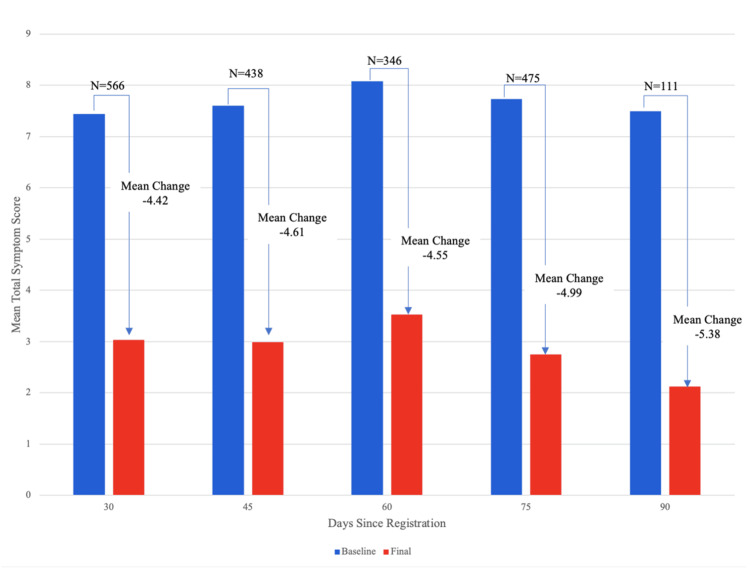
Change in the total symptom score over time The total symptom score is on a scale from 0 to 36 (a sum of 0 to 4 across nine symptoms). All mean changes in total symptom scores were statistically significant (P < 0.001).

The mean change in total symptom score was -4.7 (SD = 4.7); this represents a 61% decrease in symptom severity across the population. The total number of days of engagement with the program was significantly associated with users’ change in total symptoms score (P = 0.02, Figure 3), with participants demonstrating significantly decreased symptoms as early as day 30 post-registration.

At the end of the intervention, 1652 (85.3%) participants improved, 113 (5.8%) had no change, and 171 (8.8%) worsened. Participants with Crohn’s Disease (P = 0.004), gastroparesis (P = 0.03), GERD (P = 0.02), and IBS (P < 0.001) had statistically significant changes in total symptom scores at the end of the intervention.

Women had a higher baseline total symptom score (mean = 8.0, SD = 4.6) than men (mean = 6.3, SD = 4.4) (P < 0.001). They also had a larger change in total symptom scores at the end of the intervention (-5.1 vs. -4.1 for men, P < 0.001). At the end of the intervention, women had significantly greater improvements in constipation, gas, nausea, and bloating (P < 0.05 for all); however, there were no gender differences in the improvements observed for the other five GI symptoms.

Participants of all races experienced significant improvements in their mean total symptom score. The small population of Native American participants (N = 6) had both the highest baseline total symptom score (mean = 10.7, SD = 6.6 compared to 7.6, and SD = 4.6 for the whole population) and the largest improvement in their GI symptoms (mean change for Native Americans = -7.5, SD = 5.1 compared to -4.8, and SD = 4.5 for the whole population). Participants who scheduled at least one visit with their care team after 30 days had a greater improvement in their total symptom score than participants who did not (mean change = -5.0 vs -4.1, P < 0.001). Similarly, participants who logged their symptoms on more than two occasions had a greater improvement in their total symptom score than participants who did not (mean change = -5.5 vs -3.9, P < 0.001).

Symptom Severity

**Figure 4:**
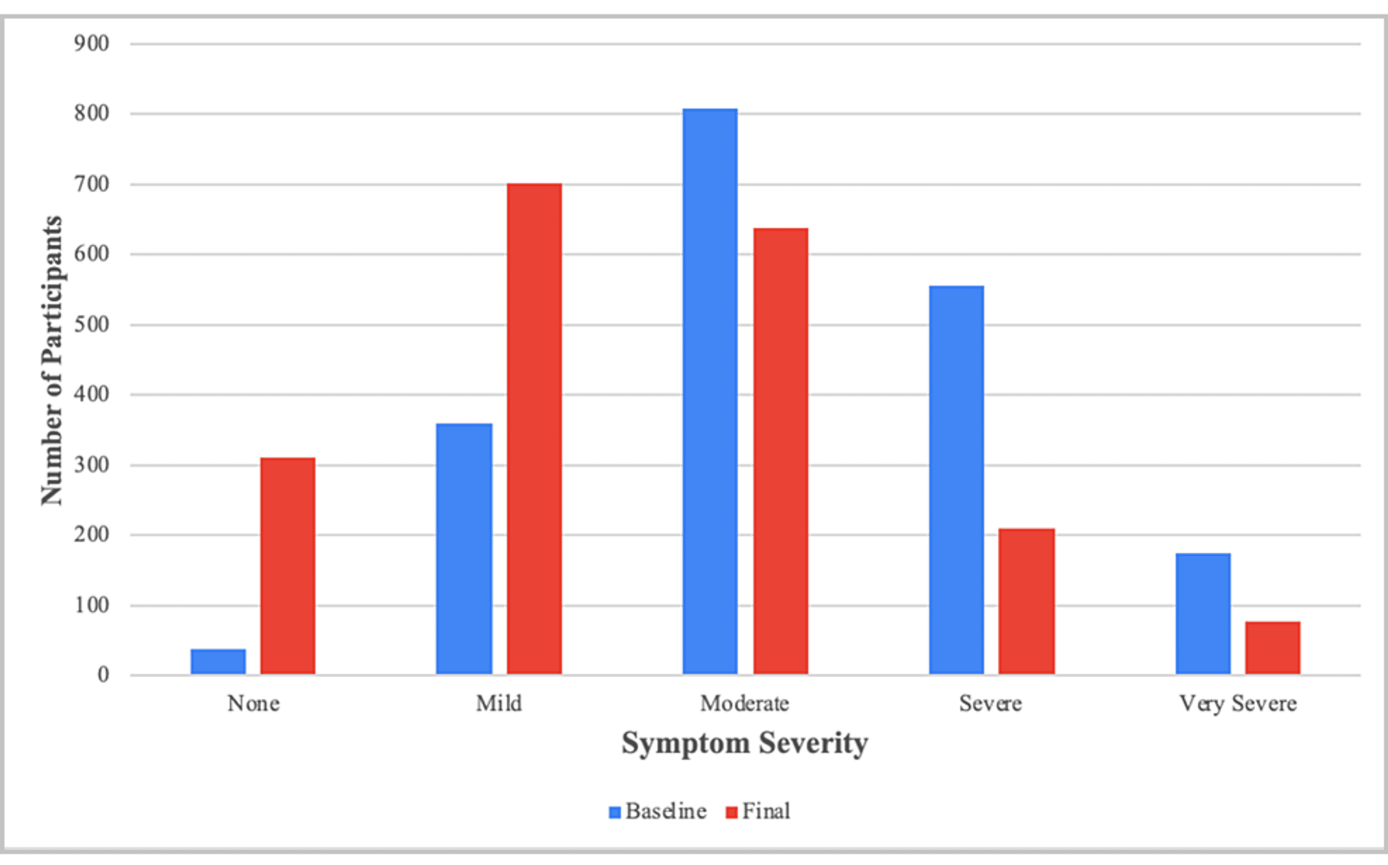
Change in the symptom severity

At baseline, 79.5% of participants reported at least moderate GI symptom severity for at least one symptom (Figure 4).

In contrast, only 47.8% of participants reported moderate or severe symptoms at the end of the intervention. Notably, at the end of the intervention, 310 (16.0%) participants reported no symptoms.

Patient-reported outcomes

Five hundred and thirty-two participants (27.4%) completed the patient-reported outcomes survey. Of these, 83% endorsed, “Since joining the program, I feel more in control of my health;” 76% endorsed, “I’m happier since using the program;” 54% endorsed, “Since joining the program, I feel more productive at work;” and 83% endorsed “The program has helped me better manage my digestive symptoms.” When asked the question, “Where would you have gone for digestive health care if you had not signed up for the program?”, 186 responded that they would not have received care, 183 would have gone to a primary care physician, 76 would have gone to a gastroenterologist, three would have gone to the emergency room, two would have gone to urgent care, and 38 would have sought other care.

## Discussion

This study of a novel digital digestive chronic disease management program for a demographically diverse population demonstrated four key findings. First, adults of a wide range of ages, genders, and races engaged with digitally enabled disease management for digestive symptoms. This is an important finding since women and non-white populations have higher rates of some GI symptoms but historically had lower rates of screening for key GI conditions (e.g., colorectal cancer and GERD) and poorer outcomes [14-17]. This suggests that the GI care management program recruiting materials were appealing and relevant to users with GI symptoms from a wide range of racial groups and could contribute to ongoing efforts to increase access to GI specialty care for vulnerable populations.

Second, the simple GI symptom tracking tool, which asked participants to rate nine common GI symptoms on a scale from none to very severe, seemed acceptable to users who used it to report symptoms over the three-month study interval. The field lacks consensus regarding the best instruments for tracking GI complaints, especially for use in virtual care settings, and many of the available tools are either long [18,19] or only relevant for a limited number of GI conditions [7,8,20]. The digital symptom tracker developed for this care management program holds promise for addressing this gap in the literature. A study that validates this tool in comparison to established, especially GI condition-specific measures, is a research priority.

Third, participation in the disease management program was associated with a significant reduction in GI symptoms even as early as day 30 post-registration. At baseline, the mean total symptom score was 7.7 (SD = 4.7), which improved at the end of the intervention to 3.0 (SD = 3.2, P < 0.001). At baseline, 80% of participants reported at least moderate GI symptom severity for at least one symptom, and by the end of the intervention, only 48% of participants reported such symptoms. These data suggest that when participants’ symptoms resolved (or improved dramatically), they stopped tracking symptoms. Moreover, a digitally enabled GI care management program seems to be an effective approach for mitigating common, debilitating GI symptoms. Importantly, GI symptoms of participants of all races/ethnic groups improved. Given health disparities in GI treatment and outcomes [14-16], a detailed evaluation of outcomes beyond symptom improvement by race/ethnicity is warranted.

Participants who were scheduled with their care team reported greater symptom improvement than those who were not scheduled. This underscores the importance of health coaching and medical nutrition therapy, as demonstrated elsewhere [21]. Notably, participants who used the app for a longer duration and logged more symptoms experienced greater improvements in their symptoms. This suggests that digital care management programs might benefit from strategies to encourage these interactions, such as digital reminders, outreach from the care team, and incentives.

Finally, in addition to symptom reduction, participants reported other important subjective outcomes, including feeling greater control of their health (83%), better able to manage their digestive symptoms (83%), increased happiness (76%), and greater productivity at work (54%). The magnitude of these self-reported effects is large compared to other workplace interventions [22]. All of these findings warrant further evaluation with validated tools for the assessment of health-related quality of life [23], depression screens [24], and workplace absenteeism and presenteeism [25].

This study had three key limitations. First, this study did not have a control group, and our statistical power was limited by the number of participants who completed the surveys at all time points. A controlled trial that compares participation in this GI disease management program to other related interventions would be a valuable contribution to the literature. A key finding of this analysis was that participants with the most baseline symptoms and those who used the care team had greater improvement in symptoms. This might represent regression to the mean and warrants further evaluation with a control group. Relatedly, we did not have an opportunity to control for confounding bias. For example, we did not assess the types of additional care (e.g., no additional care, primary care only, and specialty care) that participants might have been receiving. A controlled evaluation should consider the contribution of such additional interventions. Second, the study population included only commercially insured adults. This limits the generalizability of these results to other populations, including adolescents, the elderly, and those with other types of health insurance. Finally, all the condition data were self-reported and were not corroborated with diagnoses from medical records or claims.

## Conclusions

Given the challenges in accessing GI specialty care, a digital digestive chronic care program offers a promising tool for increasing access for populations with chronic GI symptoms, especially those with historically poorer access to GI care. The results of this study underscore the importance of incorporating GI dietitians and health coaches. Further research is urgently required to assess the ability of virtual care programs to engage and provide effective digestive care over the long term.
